# Transient Regression of Breast Carcinoma After Diagnostic Biopsy and Tumor Heterogeneity: A Case Report

**DOI:** 10.7759/cureus.72919

**Published:** 2024-11-03

**Authors:** Eduardo De Faria Castro Fleury, Jose Augusto de Sousa Neto, Sheila Cristina L Wludarski, Edson M Barbosa

**Affiliations:** 1 Radiology, MD duFLE Diagnósticos, São Paulo, BRA; 2 Mastology, Instituto Brasileiro de Controle do Câncer, São Paulo, BRA; 3 Pathology, Instituto Hermes Pardini, Grupo Fleury, Sao Paulo, BRA; 4 Pathology, Hospital Sirio Libanês, São Paulo, BRA

**Keywords:** histologic remission, immunogenic cell death (icd), primary breast malignancy, spontaneous regression of cancer, tumor heterogeneity

## Abstract

Spontaneous cancer regression is a rare biological phenomenon; however, the mechanisms involved in this process are still poorly understood. We report the case of a 92-year-old woman with a histological diagnosis of breast carcinoma. On the date of the scheduled surgical resection of her tumor, she did not present the initial lesion diagnosed by biopsy, on clinical examination.

The patient underwent mammography and breast magnetic resonance imaging, which confirmed the absence of the initial tumor. Due to religious beliefs, the patient refused additional specific treatment with her family's approval. In consensus with her family, we proposed imaging follow-up to assess tumor recurrence, which lasted for 18 months.

In this article, we discuss the theory that the local trauma generated by the diagnostic percutaneous biopsy triggered an immune response responsible for the phenomenon of tumor regression. The secondary objective is to discuss the transformation in the histological type of tumor cell.

## Introduction

Spontaneous regression of breast cancer is a rare and intriguing phenomenon that poses a significant challenge to understanding the biological mechanisms involved. Although the causes of this phenomenon are not fully understood, we know that tumor regression occurs due to the host's immune response to the tumor cells or their byproducts. Spontaneous regression is the partial or complete regression of the primary tumor and the associated metastases in the absence of specific treatment or with therapy considered inadequate to influence the evolution of the neoplastic disease [1-4].

Tumor regression has been recorded in several types of human cancer, including breast cancer, testicular germ cell tumors, neuroblastoma, melanoma, bladder cancer, kidney tumors, leukemia, lymphoma, and tumor metastasis [1]. Some biological processes are considered tumor regression trigger points, especially those related to endocrine influences, inflammatory processes, necrosis, local trauma, fevers, acute infections, reduced vascular supply, and immune system activation [5].

Several studies aim to clarify the tumor regression mechanism of HER2-positive tumors undergoing neoadjuvant therapy, given their specific immunological aspect [6]. A mechanism can inactivate tumor cell growth or death with the opposite action throughout the oncogenic promotion [7-9]. In the biological diversity found amid tumor heterogeneity, dominant clones may prevail. For this reason, even in cases of complete spontaneous regression, we cannot consider the case a cure. Most of the time, the natural selection of resistant cell subclones may result in neoplasm recurrence [7].

## Case presentation

**Figure 1:**
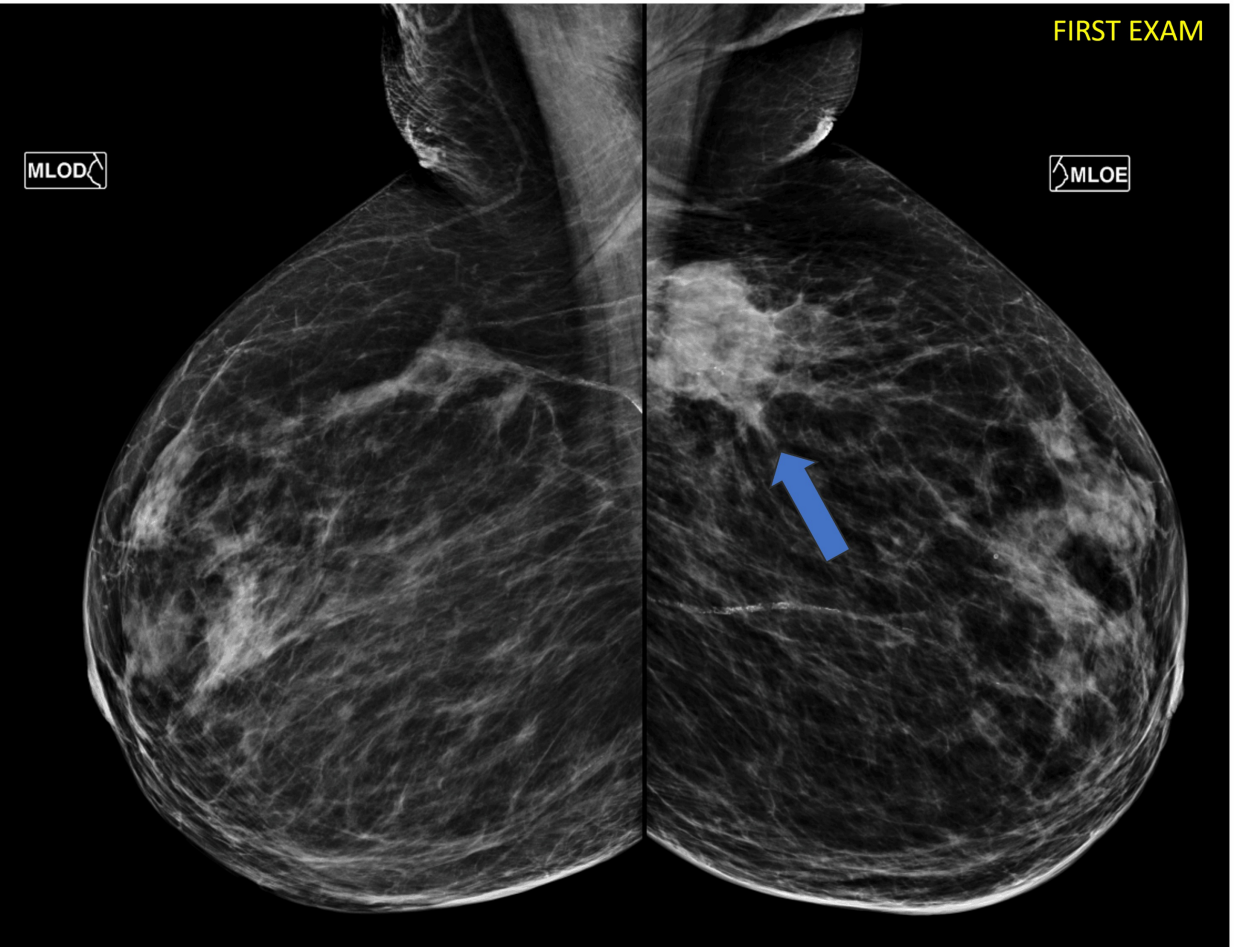
First diagnostic mammography. There is a spiculated dense mass in the left breast associated with suspicious calcifications (arrow).

A 92-year-old woman with no personal or family history of cancer was referred to the Department of Breast Surgery of the Brazilian Institute for Cancer Control (IBCC/Hospital São Camilo Oncology), reporting a rapidly growing palpable mass in her left breast for four months. On physical examination, she had a thickened palpable mass measuring 5.0 cm adherent to the deep plane in the superior lateral quadrant of the left breast without associated findings. She was referred for diagnostic mammography. According to the Breast Imaging Reporting & Data System (BI-RADS) lexicon, mammographic images showed a dense mass associated with suspicious calcifications, classified as category 5 (Figure 1).

The patient underwent a percutaneous core biopsy five days after the mammographic examination. The pathological diagnosis confirmed an undifferentiated carcinoma of the breast.

**Figure 2:**
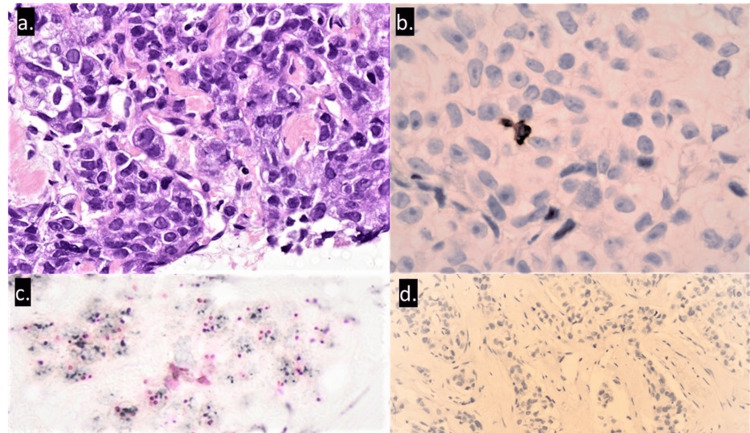
H&E staining shows a poorly differentiated invasive carcinoma of the breast not otherwise specified. (a) H&E staining shows a poorly differentiated invasive carcinoma of the breast not otherwise specified, with < 1% of stromal tumor-infiltrating lymphocytes; (b) immunohistochemical staining shows very rare CD4-positive lymphocytes, <1%; (c) HER2 Dual In Situ Hybridization was positive for HER2 amplification (HER2 gene average signals 4.0 and Chr17 average signals 2.0, with ratio of 2.0); (d) programmed cell death ligand 1 (PD-L1) assay (SP142, Ventana, Tucson, AZ, USA) was negative (immune cell (IC) score 0%).

We proposed a surgical resection of the residual area of the primary tumor. The histological findings of the biopsied lesion showed a poorly differentiated invasive carcinoma of the breast not otherwise specified (NOS; WHO 2019), with <1% of stromal tumor-infiltrating lymphocytes (TILs). The denominator used to determine the percentage of stromal TILs is the area of stromal tissue (i.e. area occupied by mononuclear inflammatory cells over total intratumoral stromal area), not the number of stromal cells. Immunohistochemical results indicate the expression of pan-cytokeratin (AE1/AE3, Ventana) and SOX10 (clone SP267, Ventana) by tumor cells, confirming breast carcinoma. Estrogen (clone SP1, Ventana) and progesterone receptors (clone 1E2, Ventana) were negative, and HER2 (clone 4B5, Ventana) was scored 2+. Ki-67 (clone 30-9, Ventana) labeling index was 80% at an eyeballing estimate. Very rare lymphocytes (<1%) expressed CD4 (clone SP35, Ventana), CD8 (clone SP57, Ventana), CD3 (clone 2GV6, Ventana) and CD20 (clone L26, Ventana). The HER2 Dual in Situ Hybridization (Ventana) was positive for HER2 amplification (Figure 4). The programmed cell death ligand 1 (PD-L1) test (SP142, Ventana) resulted in a negative result (immune cell (IC) score 0%) (Figure 2). PD-L1 was performed for research/academic interest only; the patient or her insurance was not billed. Invasive carcinoma of the breast not otherwise specified, with HER2 Dual In Situ Hybridization positive for HER2 amplification (HER2 gene average signals 4.0 and Chr17 average signals 2.0, with a ratio of 2.0).

**Figure 3:**
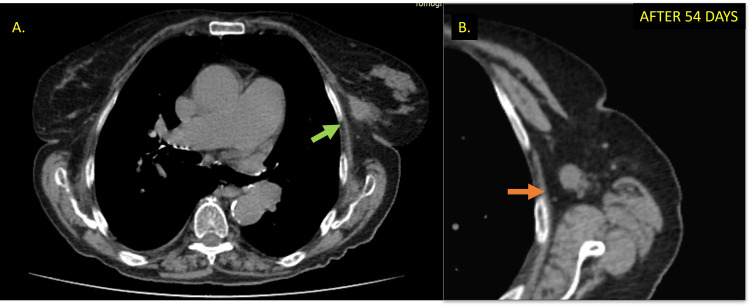
Chest computed tomography after the percutaneous biopsy. The mass described in the mammography (A) showed a reduction in dimension (green arrow). An enlargement and atypical lymph (B) node is observed in the axilla (orange arrow).

The patient returned after 35 days, reporting a significant reduction in the mass tumor size, which was confirmed on clinical examination. An additional chest computed tomography (CT) was performed to assess the extent of tumor for staging. The CT images revealed tumor involvement in the muscle plane and a reduction in the size of the tumor mass. The CT also showed an enlarged lymph node (Figure 3).

After 66 days, the patient reported that she no longer noticed the tumor mass on self-palpation in a new follow-up appointment. New clinical examinations, mammography, and MRI were performed for documentation. The palpatory clinical examination revealed total regression of the tumor and the absence of compromised lymph nodes in the axilla.

**Figure 4:**
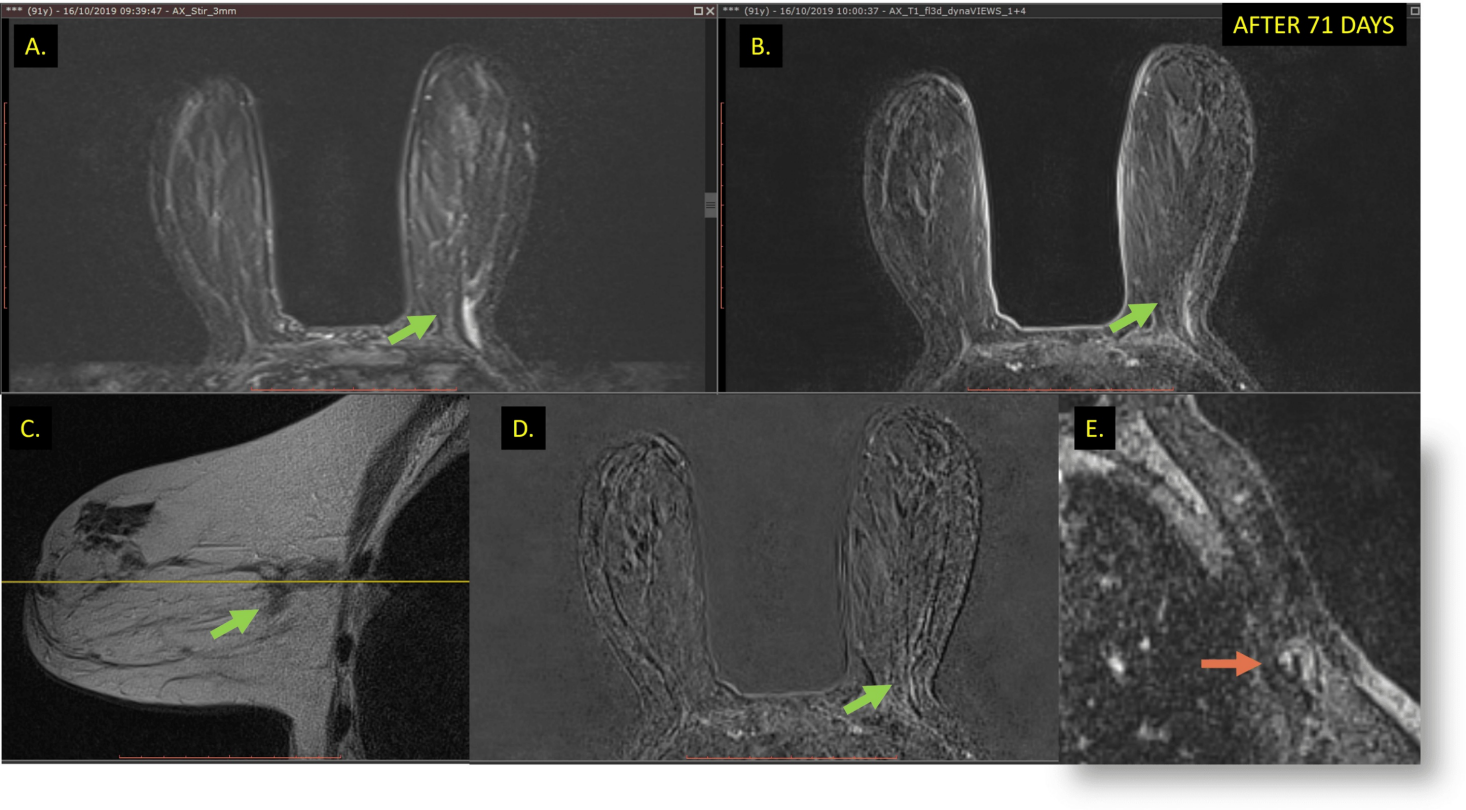
Breast MRI after biopsy. Breast MRI showing tumor remission compatible with a complete radiological response (green arrow) (A, B, C and D) (orange arrow) and lymph node (E) (orange arrow) regression.

The new mammography images performed 71 days after percutaneous biopsy showed complete radiological regression of the primary mass. A suspicious focus of calcifications remained at the mass site. According to the Breast Imaging and Reporting Data System (BI-RADS^TM^) lexicon, the mammographic classification was Category 6. The magnetic resonance imaging (MRI) confirmed the absence of an enhancement mass with mild architectural distortion at the biopsy site, which is compatible with fibrous tissue. In analogy to the criterion of tumor response to neoadjuvant treatment, the analysis considered a complete radiological response (Figure 4).

The patient rejected the surgical treatment, believing that she experienced spiritual healing, opting for clinical follow-up without any specific treatment. Surgical treatment was then authorized and the patient underwent modified left radical mastectomy and ipsilateral axillary lymph node resection. 

**Figure 5:**
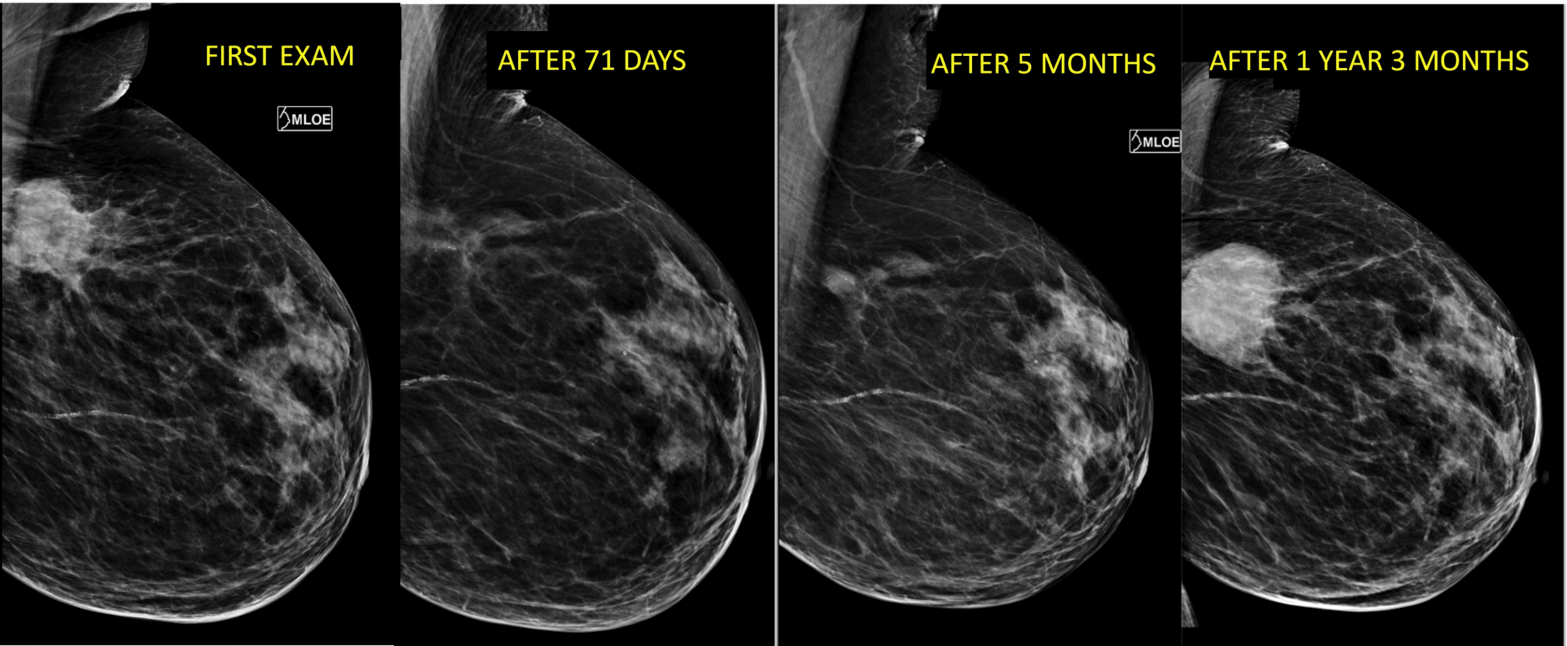
Evolutionary mammographic control. Evolutionary mammographic control showing tumor growth and regression during the follow-up.

Five months later, the mammographic control showed the recurrence of a small density in the primary tumor site. The images showed suspected calcifications associated with the mass. After one year and three months of treatment refusal, the patient noticed a growing mass at the tumor site. On clinical examination, we observed a new tumor in the superior lateral quadrant of the left breast measuring 4.0 cm. These findings were confirmed by the mammographic test (Figure 5). The axilla was free from lymph nodes showing characteristic features of metastasis radiographically. 

Quadrantectomy and axillary lymph node sampling (level one) were performed. Tumor macroscopy showed a white, well-circumscribed mass with a 4.0 cm diameter. Histology revealed a poorly differentiated invasive carcinoma of the breast with a medullary pattern. Necrosis was extensive, and inflammatory cell infiltration was high, with lymphocytes, histiocytes, and plasma cells. TILs were estimated at 70%. The resected lymph nodes did not show metastasis.

When compared to the first biopsy, no residual primary cells were found. Immunophenotyping of inflammatory infiltrate revealed a predominance of CD3+, CD8+ (30%) and CD4+ (70%) lymphocytes (Figure 5). No CD56+ T lymphocytes were found. Small clusters of CD20+ lymphocytes were identified. The immunophenotype of the carcinoma was triple-negative: tumor cells were negative for estrogen and progesterone receptors as well as for HER2 (score 1+), and the Ki-67 labeling index was 90%. The tumor cells were positive for cytokeratin and SOX10. HER2 in situ hybridization test was negative for gene amplification. The immunohistochemical and in situ hybridization were performed using the fully automated Ventana Benchmark Ultra (Ventana, Tucson, AZ) staining system. Immunohistochemistry and in situ hybridization interpretation followed the current College of American Pathologists/American Society of Clinical Oncology (CAP/ASCO) guidelines. The PD-L1 test (SP142, Ventana) resulted in a positive IC score of 10%. Invasive carcinoma of the breast with medullary features with HER2 Dual In Situ Hybridization was negative for HER2 amplification (HER2 gene average signals 2 .0 and Chr17 average signals 2.0, with ratio of 1.0).

**Figure 6:**
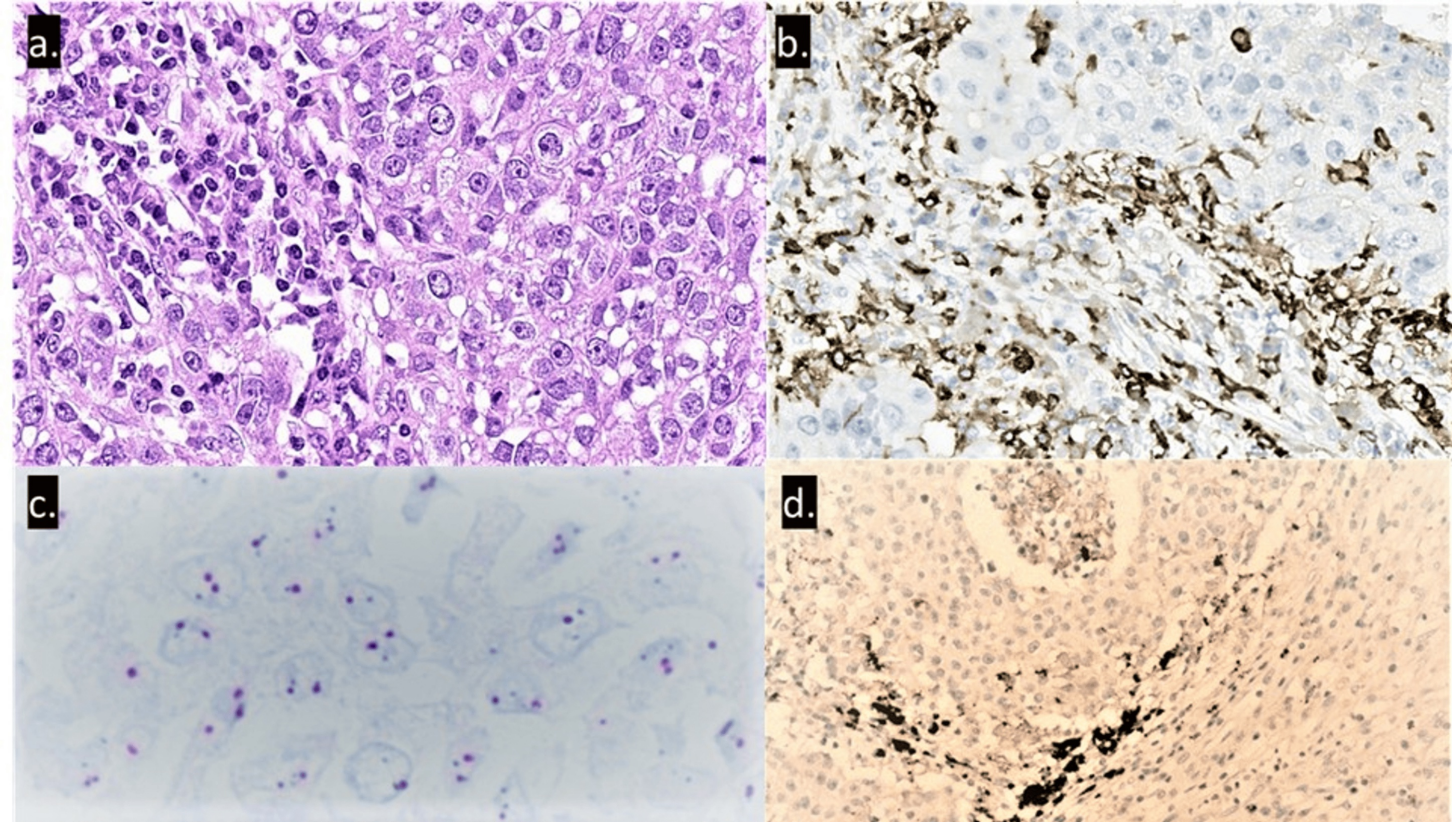
H&E staining shows a poorly differentiated invasive carcinoma of the breast with medullary features, including a rich inflammatory cell infiltration. (a) H&E staining shows a poorly differentiated invasive carcinoma of the breast with medullary features T including a rich inflammatory cell infiltration; (b) immunohistochemical staining shows a large number of CD4-positive lymphocytes, 70%; (c) HER2 Dual In Situ Hybridization was negative for HER2 amplification (HER2 gene average signals 2 .0 and Chr17 average signals 2.0, with ratio of 1.0); (d) PD-L1 test (SP142; Ventana, Tucson, AZ, USA) was positive (IC 10%).

## Discussion

Spontaneous cancer regression is an intriguing and challenging phenomenon frequently reported in the medical literature but still poorly understood. It has unknown mechanisms involved, certainly motivated by the scarcity of evidence. It is well demonstrated that this phenomenon can occur in almost all types of tumors, whether benign or malignant, primary or metastatic [1-3].

For a neoplastic cell to become competent, progress, and spread, a cascade of genetic messages must be transmitted, received, and retransmitted through different signaling pathways in an interactive way between tumor cells, their microenvironment, extracellular matrix, and the macro-environment of the host. The cross-relationship between cells interferes with the microenvironment remodeling, facilitating tumor progression [1,4].

Simultaneously with the proliferation of neoplastic cells, the body develops barriers to tumor growth by the immune system. The disease progresses when the aggressor defeats the defense. Interferences in this cellular adequacy system can interrupt the proliferation and replication processes and stimulate opposite commands for damage repair, leading to cellular destruction [5,7].

Some known mechanisms are responsible for these interferences. The mechanisms include genetic and epigenetic commands, immunological mediations, neuroendocrine and hormonal interactions, inhibition of growth factors and/or cytokines, induction of cell differentiation, necrosis stimulation, inhibition of angiogenesis, trauma, and infections [8,9]. The possibility that these processes can act as inducers of cell destruction is well established. At least in some cases, they could be responsible for the spontaneous regression (complete or temporary) of breast cancer [8,9].

Since malignant tumors are characterized by genomic, phenotypic, and antigenic diversities, we can infer that lethal damage may be selective, explaining cell modulation during tumor evolution [10,11]. We should also emphasize that, in the relationship between the immune system and the tumor, innate immunity composed of natural killer cells, eosinophils, basophils, and phagocytic cells, is dominant mainly over adaptive immunity integrated by B, T, CD4, and CD8 lymphocytes. Or whether the antigen recognition of specific cellular protein molecules overcomes the action of antibodies activated by the adaptive immune system [12].

Infiltrating lymphocytes correspond to important prognostic factors and predictors of therapeutic response, especially in HER2(+) and triple-negative tumor subtypes. The increased detection of these cells in the tumor environment suggests an anti-tumor immunogenic effect, whose efficacy depends on its diversity, specificity, and dysfunction shaped by the immunosuppressive microenvironment [13-15].

Immune prediction responses mediated by proteins expressed by T-cell (CD8), natural killer (NK), and dendritic cells inhibitory characteristics have also been confirmed in studies with monoclonal antibodies directed to block other inhibitory signal proteins at the immunological checkpoint. Thus, PD-1 expressed on the surface of T cells binds to PD-L1 and exhaustively activates T cells, inducing cytotoxicity and the mechanism of programmed tumor death [16]. These described mechanisms are evident in the responses to neoadjuvant treatment, especially in tumors with HER2 expression [13].

Based on these observations, we adopted the theory proposed by Dussan et al. and Radha et al. that diagnostic percutaneous biopsy can trigger a disturbance of the tumor microenvironment balance. The local trauma should cause the release of antigens by tumor cells and a subsequent local inflammatory process, which stimulates the immune system and activates the planned cell death command (apoptosis) [17]. Our hypothesis corroborates the study published by Tokunaga et al., which infers that the spontaneous response of induced T cells corresponds to the initial signaling mechanism of spontaneous tumor eradication [4,16].

Although some studies discussed the interference of the role of certain drugs in tumor regression often used as conservative therapy in the setting of ductal carcinoma in situ (DCIS), such as metformin and statins, it was impossible to establish any correlation with these drugs in the current report. The patient did not experience any medication for continuous use or chronic diseases [1].

Following the theory of tumor heterogeneity, we believe that the immunological system acted on specific cells, which were dominant in the composition of the primary tumor. In this context, there was a natural selection of neoplastic cell lines in the tumor microenvironment. Added to this, we cite the residual inflammatory process resulting from the first response, which may have provided a fertile environment for the proliferation of the other histological lineage.

Another hypothesis for changing cell lines can be credited to chronic exposure of epithelial cells to aggressive stimuli in the tumor microenvironment, favoring cell dysplasia as has been relatively well documented in the step-wise progression of epithelial tumors of the adnexa (fallopian tube-ovary) [18,19]. As there was no surgical tumoral excision, the mechanisms described can explain the reappearance of a new cell lineage, reinforcing the need for surgical excision even in cases of complete response to drug treatment.

## Conclusions

Drawing inferences about the complex biological mechanisms that govern spontaneous tumor regression is challenging. We discuss the theory that the local trauma generated by the diagnostic percutaneous biopsy triggered an immune response responsible for the phenomenon of tumor regression.

Better knowledge of these processes may help us decipher this interesting natural effect, its potential applications to patient management (as in when to avoid overtreatment), or how to leverage this response to tailor specific therapies to tumor types, such as medullary carcinoma.
